# Multi-omics mapping identifies CYBA-mediated mitochondrial dysfunction driving macrophage polarization and ferroptosis via Nrf2 pathway in atherosclerosis

**DOI:** 10.1063/5.0303714

**Published:** 2026-01-30

**Authors:** Chen Dong, Rui Shen, Chengliang Pan, Jiangmei Zhang, Kunwu Yu, Qiutang Zeng

**Affiliations:** 1 Department of Cardiology, Union Hospital, Tongji Medical College, Huazhong University of Science and Technology, Wuhan 430022, China; 2 Hubei Key Laboratory of Biological Targeted Therapy, Union Hospital, Tongji Medical College, Huazhong University of Science and Technology, Wuhan 430022, China; 3 Hubei Provincial Engineering Research Center for Immunological Diagnosis and Therapy for Cardiovascular Diseases, Union Hospital, Tongji Medical College, Huazhong University of Science and Technology, Wuhan 430022, China

## Abstract

Atherosclerosis (AS), a chronic inflammatory process driven largely by macrophage-mediated plaque formation, remains poorly understood in mitochondrial–macrophage crosstalk. While CYBA polymorphisms correlate with cardiovascular risk, the functional role of CYBA in connecting mitochondrial dysfunction to macrophage phenotypic alteration and functional modulation remains largely unknown. In this study, we integrated multi-omics profiling of AS immune microenvironments with mitochondrial-associated gene sets. Machine learning and single-cell RNA sequencing identified CYBA as a key oxidative stress regulator. CYBA expression was significantly upregulated both in oxidized low-density lipoprotein (ox-LDL)-stimulated THP-1 macrophages and in atherosclerotic lesions, with immunofluorescence confirming macrophage enrichment. *In vivo*, ApoE^−/−^ mice fed a high-fat/high-cholesterol diet and adeno-associated virus-mediated CYBA knockdown attenuated atherosclerotic plaque formation and lipid deposition and rescued mitochondrial damage. *In vitro*, CYBA silencing attenuated ox-LDL-induced mitochondrial dysfunction and oxidative stress, concurrently inhibiting pro-inflammatory polarization and ferroptosis. Mechanistically, CYBA deficiency facilitated Nrf2 nuclear translocation and downstream activation of heme oxygenase 1 and NAD(P)H quinone dehydrogenase 1, whereas pharmacological Nrf2 inhibition reversed these protective effects. Our findings unveil CYBA as a mitochondrial checkpoint that constrains Nrf2-mediated antioxidant responses, thereby promoting inflammatory polarization and ferroptosis in macrophages during AS. Targeting the CYBA offers a promising therapeutic strategy to attenuate plaque progression.

## I. INTRODUCTION

Atherosclerosis (AS), a chronic inflammatory disorder underlying coronary heart disease and stroke, remains a leading cause of global cardiovascular mortality despite advances in lipid-lowering therapies and interventional procedures. This therapeutic impasse highlights the urgent need to elucidate novel pathogenic mechanisms, particularly those involving immune–metabolic dysregulation within atherosclerotic plaques. Macrophages, as central orchestrators of plaque inflammation, exhibit remarkable functional plasticity that dictates disease progression.^1^

Mitochondria serve as the cellular powerhouses responsible for oxidative phosphorylation and adenosine 5′-triphosphate (ATP) synthesis, playing a key role in regulating macrophage function through energy metabolism, transcriptional regulation, and signaling cascades.^2^ Under physiological conditions, mitochondrial respiratory chains generate low levels of reactive oxygen species (ROS), which are counterbalanced by antioxidant systems such as the glutathione (GSH) pathway. In atherosclerosis, however, oxidized low-density lipoprotein (ox-LDL) disrupts this equilibrium by inducing pathological ROS overproduction, triggering mitochondrial membrane potential collapse and electron transport chain dysfunction.^3^ The resulting oxidative stress potently activates vascular inflammatory signaling. Loss of mitochondrial membrane integrity and ROS overdrive promote pro-inflammatory NF-κB activation, driving macrophage commitment to the M1-like phenotype.^3^ Additionally, mitochondrial damage-associated molecular patterns (DAMPs) leakage induced by membrane permeabilization triggers inflammatory cascades via pattern recognition receptors.^4^ Notably, ROS overproduction from defective electron transport chains not only exacerbates oxidative stress but also drives metabolic reprogramming, favoring M1-like polarization through NF-κB activation,^5^ creating a self-sustaining cycle of inflammation. Moreover, mitochondrial ROS directly catalyze ferroptosis—a form of iron-dependent cell death marked by lipid peroxidation^6,7^—contributing to macrophage dysfunction. Ferroptosis has been implicated in diverse pathological scenarios, including myocardial ischemia–reperfusion injury and atherosclerotic foam cells.^8,9^ Ox-LDL loading primes macrophages for ferroptosis through glutathione depletion-induced glutathione peroxidase 4 (GPX4) inactivation, which impairs lipid peroxide clearance. Mitochondrial ROS further oxidize polyunsaturated fatty acid (PUFA)-enriched phospholipids, generating cytotoxic lipid peroxides that propagate macrophage death in a feed-forward loop. Atherosclerotic lesions exhibit characteristic mitochondrial ferroptotic features,^10^ underscoring the central role of mitochondrial dysfunction in coupling oxidative stress, inflammatory polarization, and programmed cell death in plaque progression.

The Nrf2/HO-1/NQO-1 signaling pathway serves as a pivotal regulator of mitochondrial homeostasis^11^ and exerts multifaceted protection in atherosclerosis. Under oxidative stress, Nrf2 dissociates from its cytoplasmic inhibitor Kelch-like ECH-associated protein 1 (KEAP1) and translocates into the nucleus, where it binds to antioxidant response elements (AREs) via heterodimerization with small Maf proteins, initiating the transcription of cytoprotective genes such as heme oxygenase 1 (HO-1) and NAD(P)H quinone dehydrogenase 1 (NQO-1).^12,13^ In macrophages, this pathway demonstrates broad immunomodulatory capacity by antagonizing NF-κB-driven pro-inflammatory polarization,^14^ thereby suppressing the transition toward pro-inflammatory M1-like phenotypes and reducing the expression of key inflammatory mediators, including IL-1β, IL-6, and CCL5.^15,16^ Crucially, Nrf2 directly transactivates GPX4 to mitigate ferroptosis by restoring glutathione (GSH)-dependent lipid peroxide detoxification.^17^ These coordinated actions highlight Nrf2's critical role as a molecular nexus in atherosclerosis pathogenesis.

Despite these insights, the systematic identification of molecular hubs linking mitochondrial dysfunction to immune dysregulation remains challenging. To address this, we integrated immune microenvironment profiling with mitochondria-focused transcriptomic analysis and cross-omics analysis, which identified CYBA as a master regulator of redox-inflammatory crosstalk in macrophages. Both *in vivo* and *in vitro* experiments demonstrated significant upregulation of CYBA under atherosclerotic conditions. These findings unveil CYBA as a novel therapeutic target that shapes macrophage phenotype and modulates function through mitochondrial mechanisms, providing a conceptual foundation for precision medicine interventions in atherosclerosis.

## II. RESULTS

### A. Exploring the immune microenvironment and immune-related genes

**FIG. 1.**
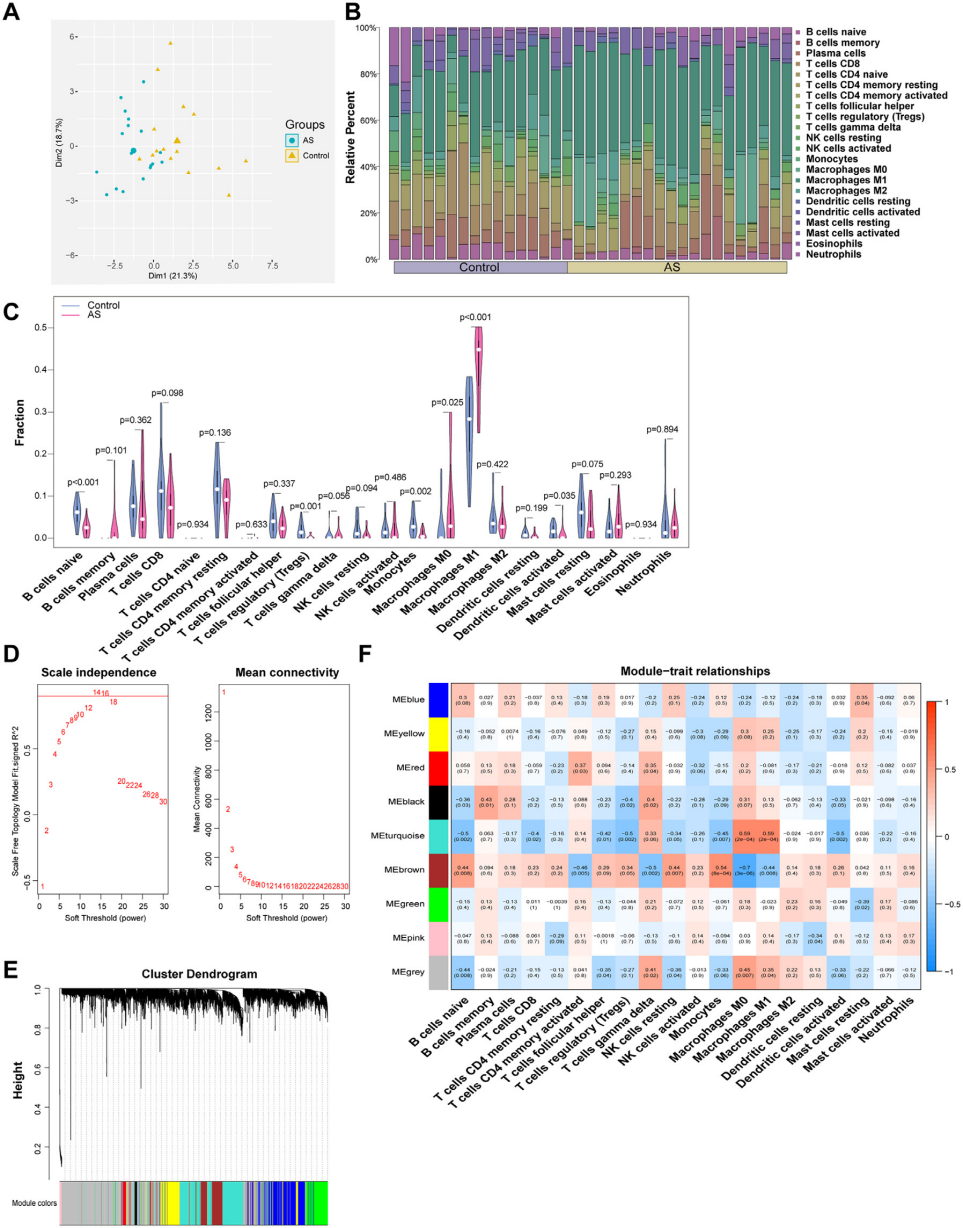
The immune infiltration analysis and immune-WGCNA analysis. (a) PCA plot comparing between AS and control groups, indicating significant differences in the immune microenvironment. (b) The proportion of 22 infiltrating immune cell types in AS and control groups. (c) Violin plots showing the distribution of infiltrating immune cells in AS and control groups. (d) Calculation of the scale-free topology index and average connectivity at different β values. (e) Dendrogram of module eigengenes, with each leaf representing an individual gene. (f) Heatmap of trait-immune cell correlations. The ME turquoise module (cor = 0.59, p = 2 × 10^−4^) and ME brown module (cor = −0.44, p = 0.008) show strong associations with M1 macrophages.

Immune cells play a crucial role in driving the chronic arterial inflammation associated with AS.^18^ The vast heterogeneity of leukocyte subpopulations in the arterial wall prompted us to conduct an immune infiltration analysis. In this study, we initially consolidated and analyzed GSE28829 and GSE226790 from the Gene Expression Omnibus (GEO) database. Principal component analysis (PCA) demonstrated a distinct separation between the AS and control groups [Fig. 1(a)]. The CIBERSORT was used to evaluate the infiltration of 22 immune cell types, with Fig. 1(b) illustrating their proportions, notably highlighting monocytes/macrophages as the predominant cell type. Six immune cells, including naïve B cells, regulatory T cells, monocytes, M0 macrophages, M1 macrophages, and activated dendritic cells, showed significant differences. Specifically, monocytes were significantly reduced in the AS group, suggesting a shift toward enhanced differentiation into plaque-associated macrophages. Meanwhile, M1 macrophages were significantly activated, indicating their involvement in the inflammation and damage of plaques, processes driven by the immune response in AS.^19^ Collectively, the results emphasize the pivotal contribution of macrophages to the progression of AS [Fig. 1(c)]. Following the construction of a scale-free network and the identification of distinct modules [Figs. 1(d) and 1(e)], we examined the correlation between these modules and infiltrating immune cells. Our analysis revealed that the ME turquoise and ME brown modules exhibited the strongest correlation with M1 macrophages. As a result, we selected a total of 1429 genes from these two modules for further study [Fig. 1(f)].

### B. Identification of mitochondrial-related genes and enrichment analysis

**FIG. 2.**
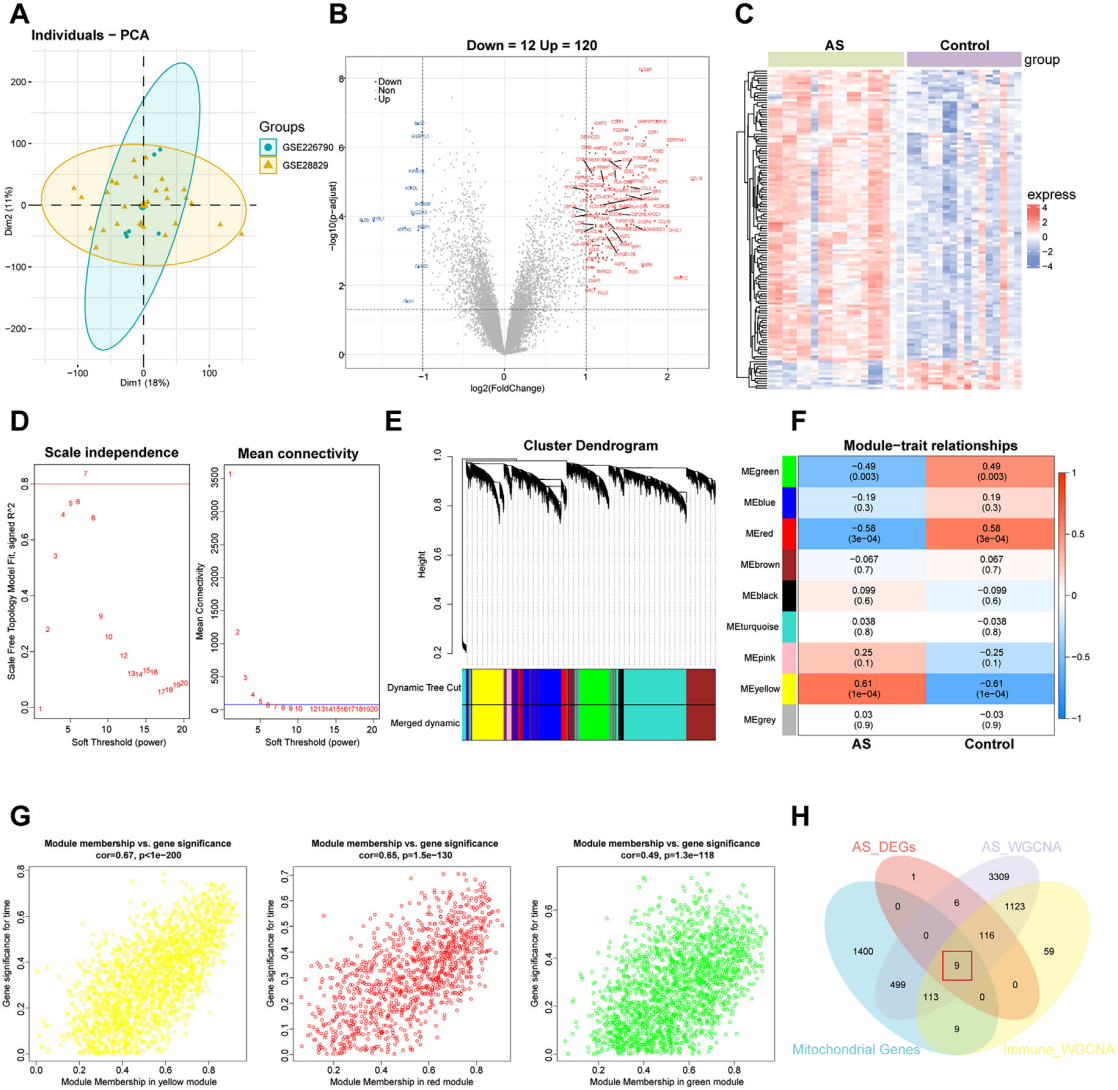
Identification of nine genes associated with mitochondria in AS. (a) PCA plots confirmed batch-effect removal, with colors denoting different datasets. (b) and (c) The volcano plot and heatmap plot of DEGs involved in AS. The red and blue colors indicate upregulation and downregulation, respectively. (d) The scale-free conformance index and average connectivity were calculated at different β values. (e) The cluster dendrogram of module eigengenes, with each leaf corresponding to an individual gene. (f) WGCNA module–trait relationships are presented, with rows indicating module eigengenes, columns representing traits, and each entry displaying the corresponding correlation and p-value. (g) Association between module membership and gene expression is plotted. Pearson's correlation analysis was performed to obtain Pearson's correlation and ME green (cor = −0.49, p = 0.003), ME red (cor = −0.58, p = 3 × 10^−4^), and ME yellow (cor = 0.61, p = 1 × 10^−4^) modules showed significant correlations. (h) Venn diagram showing the overlap between DEGs, WGCNA modules, Immune_WGCNA, and mitochondrial-related genes.

Next, to further narrow the selection, we investigated genes with altered expression in AS. We applied the criteria of |log_2_FC| ≥ 1 and adjusted p-value (p adj.) ≤0.05 for differential expression analysis. After removing batch effects through data harmonization [Fig. 2(a)]. We identified 132 differentially expressed genes (DEGs), including 120 upregulated and 12 downregulated genes. The results were visualized through a volcano plot and heatmap [Figs. 2(b) and 2(c)]. In order to delve into the functions of hub genes in AS, we conducted Gene Ontology (GO) and Kyoto Encyclopedia of Genes and Genomes (KEGG) enrichment analyses on the DEGs to uncover the potential roles in biological processes and signaling pathways (supplementary Fig. S1). We further constructed a Weighted Gene Co-expression Network Analysis (WGCNA), beginning with hierarchical clustering to select the optimal soft threshold β [Fig. 2(d)]. Using the Topological Overlap Matrix (TOM), we identified nine gene modules [Fig. 2(e)]. Notably, the ME green, ME red, and ME yellow modules showed significant correlations with AS [Fig. 2(f)]. Next, we calculated the correlation between module membership (MM) and gene significance (GS), revealing strong associations [Fig. 2(g)]. In summary, we selected 5175 genes from AS-associated WGCNA modules (AS_WGCNA), intersected them with 132 DEGs (AS_DEGs), 1429 macrophage alteration-associated genes (Immune_WGCNA) and 2030 mitochondrial-related genes from the MitoCarta3.0 and Gene Set Enrichment Analysis (GSEA) database, ultimately identifying nine candidate genes that may be involved in the regulation of macrophages mitochondrial dysfunction in AS pathogenesis [Fig. 2(h)].

### C. Hub genes selection and predictive model construction

**FIG. 3.**
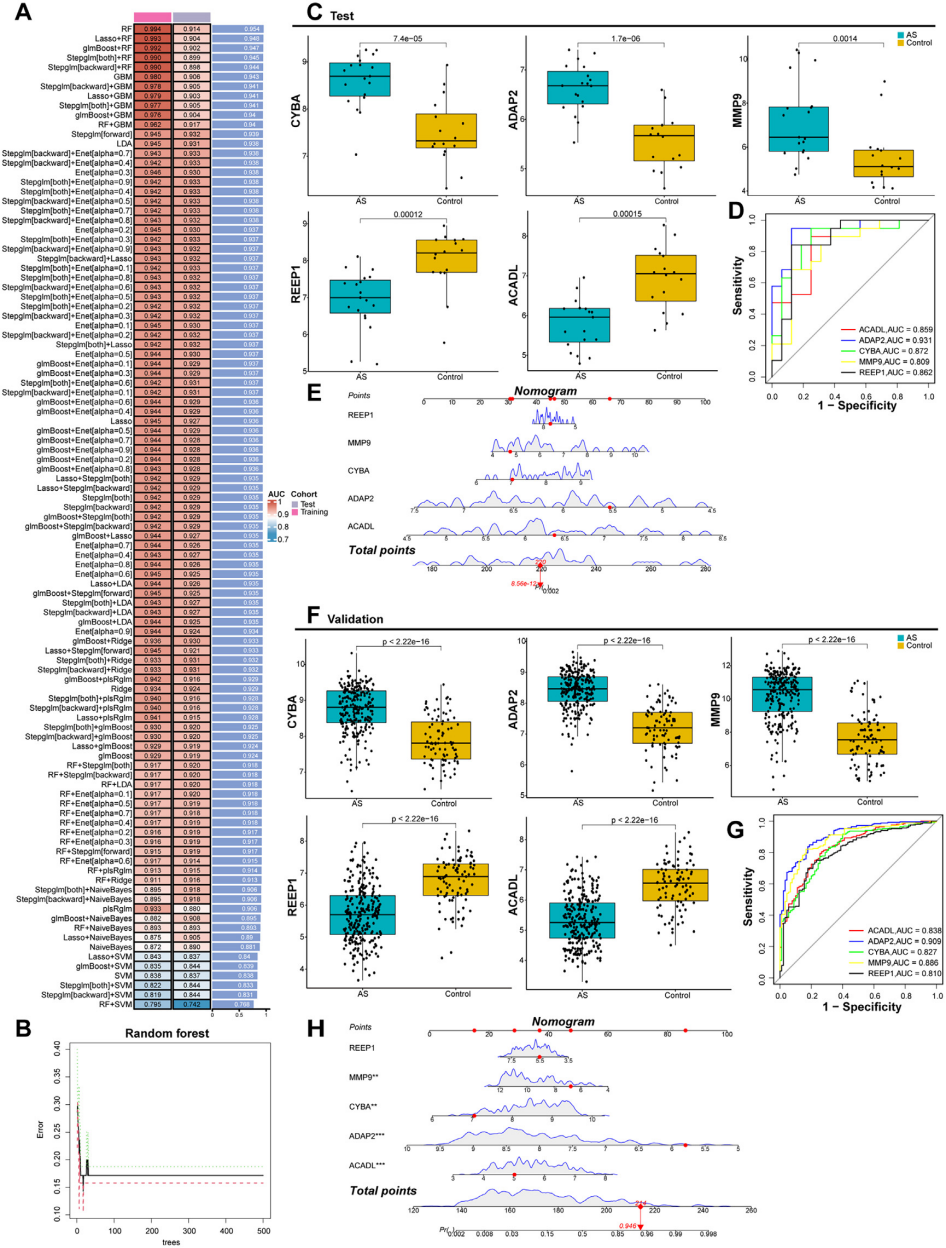
Integration of multiple machine learning algorithms to identify hub genes and construct a predictive model. (a) AUC values of 113 machine learning algorithm combinations across each cohort. (b) The connection between trees and errors in a random forest. (c) Validation of five hub genes expression between AS and control group using the test datasets (GSE28829 and GSE226790). (d) ROC curve analysis of the five hub genes in the test datasets. (e) Construction of nomogram models. (f) Validation of five hub genes between AS and control group using the validation datasets (GSE57691, GSE100297, GSE21545, and GSE43292). (g) ROC curve analysis of the five hub genes in the validation datasets. (h) Establishment of the predictive nomogram models.

Building upon the identification of nine candidate genes associated with mitochondrial dysfunction in macrophages, a total of 113 predictive models were developed utilizing machine learning integration techniques [Fig. 3(a)]. The random forest (RF) algorithm was chosen based on the area under the curve (AUC) values of these predictive models to discern characteristic genes and determine the optimal gene set [Fig. 3(b) and supplementary Table S3]. Consequently, five key genes were identified: ADAP2, CYBA, MMP9, ACADL, and REEP1. Detailed information regarding these five hub genes is provided in Supplementary Table S4. We subsequently validated the expression levels of the five target genes in both the AS and control groups, employing box plots for visualization. In the test datasets, CYBA, ADAP2, and MMP9 were significantly upregulated in the AS group, whereas REEP1 and ACADL were significantly downregulated, as indicated by statistically significant p-values [Fig. 3(c)]. To evaluate the potential predictive utility of these key gene markers in atherosclerosis, receiver operating characteristic (ROC) curves were generated for each gene within the training datasets [Fig. 3(d)]. The AUC values were as follows: ACADL (AUC = 0.859), ADAP2 (AUC = 0.931), CYBA (AUC = 0.872), MMP9 (AUC = 0.809), and REEP1 (AUC = 0.862). Additionally, a nomogram was constructed based on these five hub genes [Fig. 3(e)], illustrating their superior diagnostic performance. To further corroborate the expression of these genes, four external validation gene sets were utilized. The expression patterns observed were consistent with those in the test cohort, and ROC analysis indicated a diagnostic performance with AUC values exceeding 0.8 for all genes [Figs. 3(f)–3(h)].

### D. Protein–protein interaction (PPI), transcription factor, miRNA network, and GSEA analysis

**FIG. 4.**
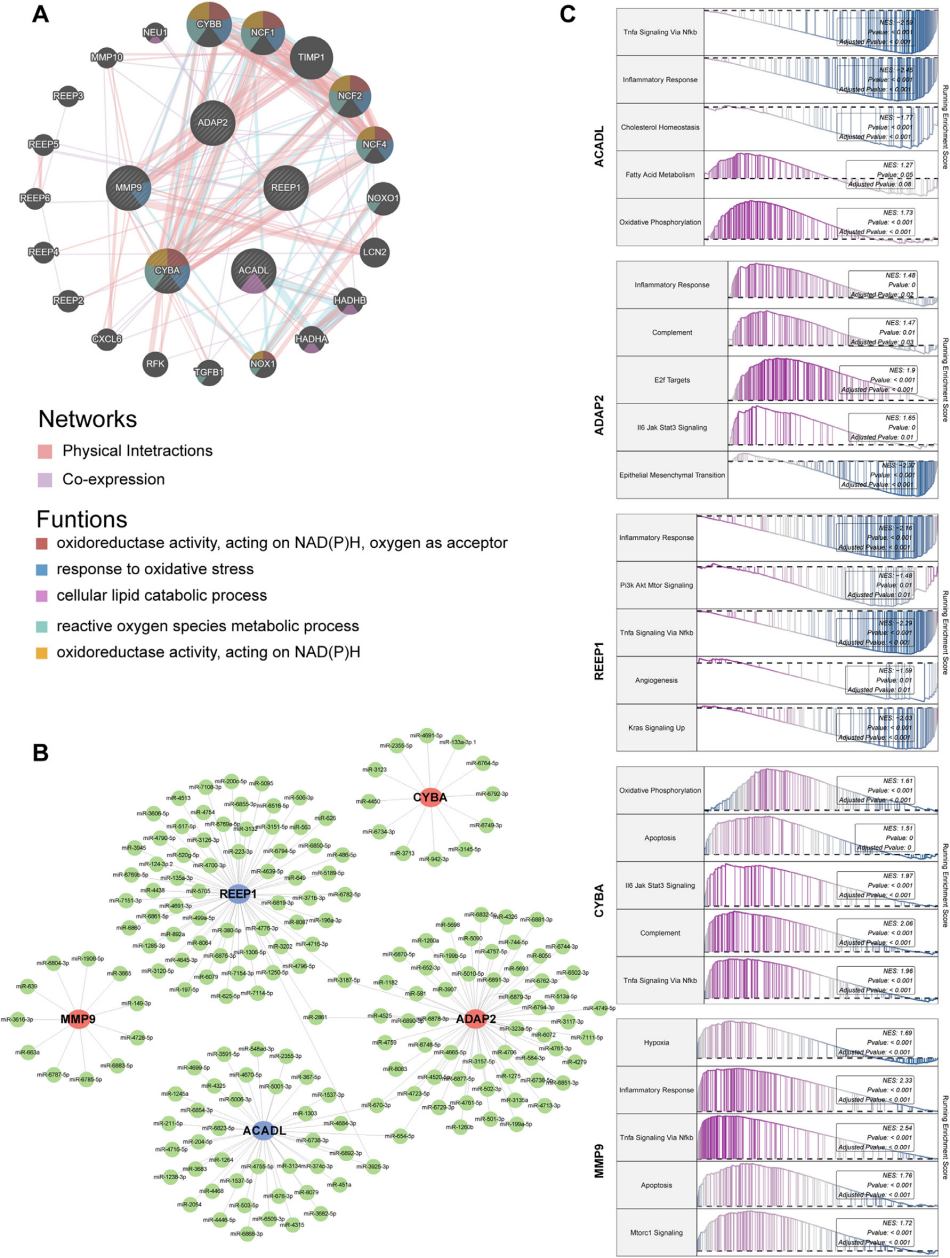
Construction of regulatory network and GSEA analysis. (a) Gene–gene interactions of five hub genes with other genes were identified. (b) miRNA-target genes regulatory network. Red nodes represent upregulated genes, and blue nodes represent downregulated genes. (c) GSEA analysis for ACADL, REEP1, CYBA, ADAP2, and MMP9 using the HALLMARK pathways as the background gene set.

Subsequently, we utilized the GeneMANIA database to construct a PPI network for the identified hub genes, which revealed a total of 20 interacting genes [Fig. 4(a)]. The PPI network analysis conducted using GeneMANIA highlighted the potential interactions among these hub genes. The results indicated that the functions of hub genes were predominantly associated with cellular response to oxidoreductase activity, acting on NAD(P)H, oxygen as acceptor, response to oxidative stress, cellular lipid catabolic process, reactive oxygen species metabolic process, and oxidoreductase activity, acting on NAD(P)H. Transcription factors (TFs) are key regulatory proteins that control gene expression by binding to specific DNA sequences, thereby coordinating cellular functions and organismal development. To elucidate the regulatory network underlying these five hub genes, we predicted the involved transcription factors using online tools KnockTF, GTRD, and ChIP_Atlas.^20^ The potential TFs were identified by intersecting the results from these three databases (supplementary Fig. S2). The regulatory network comprising miRNAs and selected DEGs associated with AS was constructed and visualized using Cytoscape. Notably, miR-3187-5p, miR-2861, among others, appear to have potential roles in regulating multiple miRNAs and their target genes [Fig. 4(b)], though further validation is needed. Subsequently, we reanalyzed the five genes based on their median expression levels within the disease cohort and performed GSEA to investigate the biological pathways potentially regulated by these molecules [Fig. 4(c)].

### E. Visualization plots of single-cell RNA sequencing in adjacent and core tissues

**FIG. 5.**
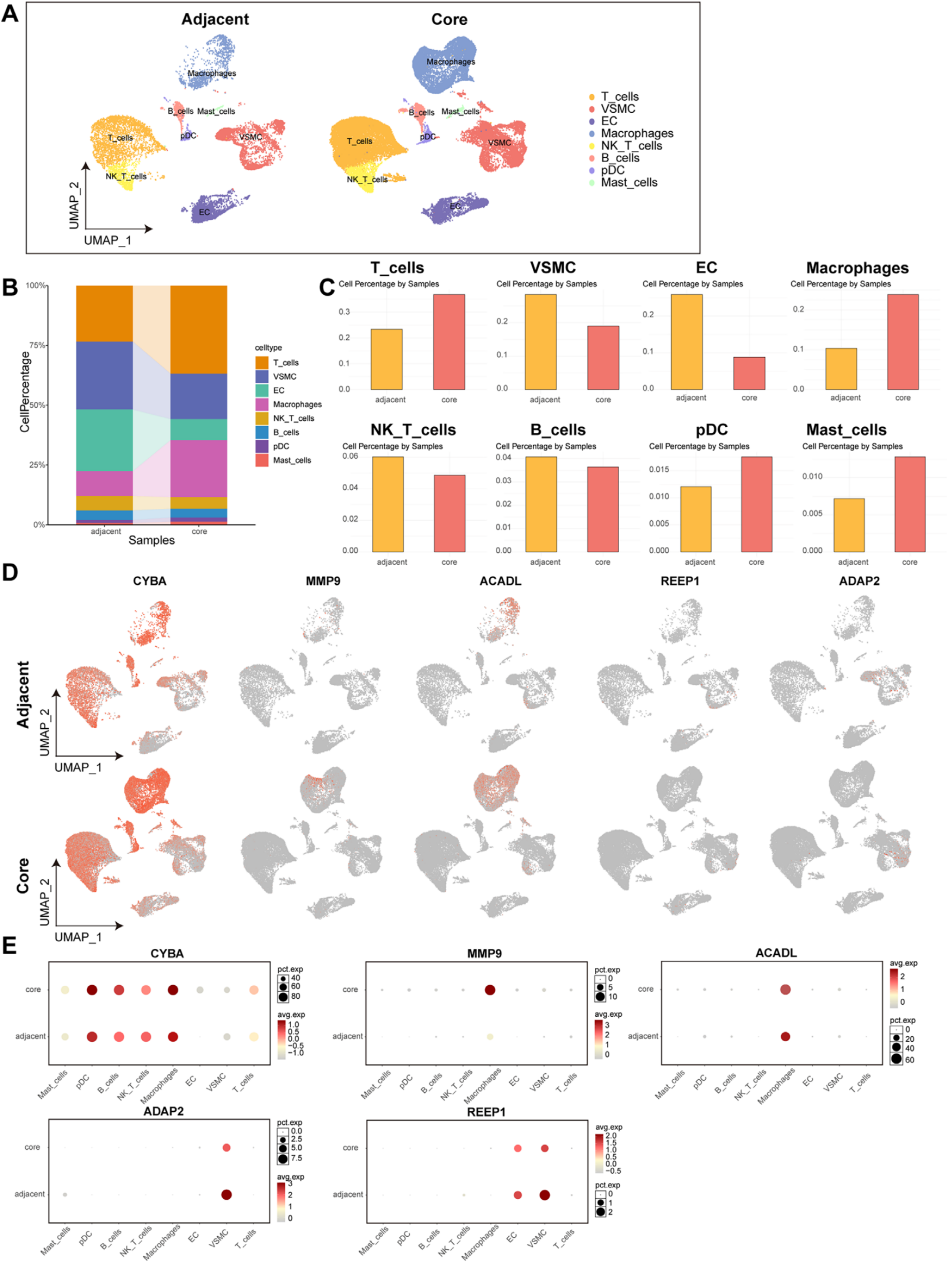
Single-cell analysis elucidates the distinct immune microenvironment with atherosclerosis. (a) Identification of cell types within the atherosclerotic core plaques and adjacent regions, with each color representing a distinct cell type. (b) and (c) Bar graphs demonstrate the proportion of each cell type. (d) The expression patterns of CYBA, MMP9, ACADL, REEP1, and ADAP2 were represented in the UMAP. (e) The gene expression levels of CYBA, MMP9, ACADL, REEP1, and ADAP2 in different cell types. Size of dot corresponds to the percentage of clustered cells and color of dot indicates the average gene expression.

We employed GSE159677 to perform single-cell analysis on three fully calcified atherosclerotic core plaques, along with patient-matched proximal adjacent regions of carotid artery tissue. Following stringent quality control (QC), ten distinct clusters were identified and assigned to various cell types [supplementary Figs. S3(a)–S3(c)], uniform manifold approximation and projection (UMAP) visualizations delineated the following cell types: T cells, vascular smooth muscle cells (VSMCs), endothelial cells (ECs), macrophages, NK_T cells, B cells, plasmacytoid dendritic cells (pDCs), and mast cells [Fig. 5(a)]. Figure 5(b) illustrates cell-specific marker genes, while Fig. 5(c) presents the distribution of each cell subtype across the samples. Within the plaques, macrophages constitute a pivotal component of the pro-inflammatory microenvironment. Our results revealed a significant increase in the proportion of macrophages within the atherosclerotic core plaques [Fig. 5(d)], implying that during atherosclerotic inflammatory injury, there is a substantial influx of monocytes infiltrating the vessel wall. This infiltration contributes to biological processes such as chemotaxis, adhesion, and phagocytosis, which are intimately associated with the formation and rupture of atherosclerotic plaques.^21^ Furthermore, we investigated the expression patterns of five hub genes using UMAP plots of the adjacent and core regions [Fig. 5(e)]. Notably, CYBA and MMP9 exhibited significantly elevated average expression levels in macrophages in the core group [Fig. 5(e)]. Conversely, REEP1 and ADAP2 were nearly undetectable in macrophages across both regions.

### F. Upregulation of CYBA and MMP9 and downregulation of ACADL *in vitro* and *in vivo*

**FIG. 6.**
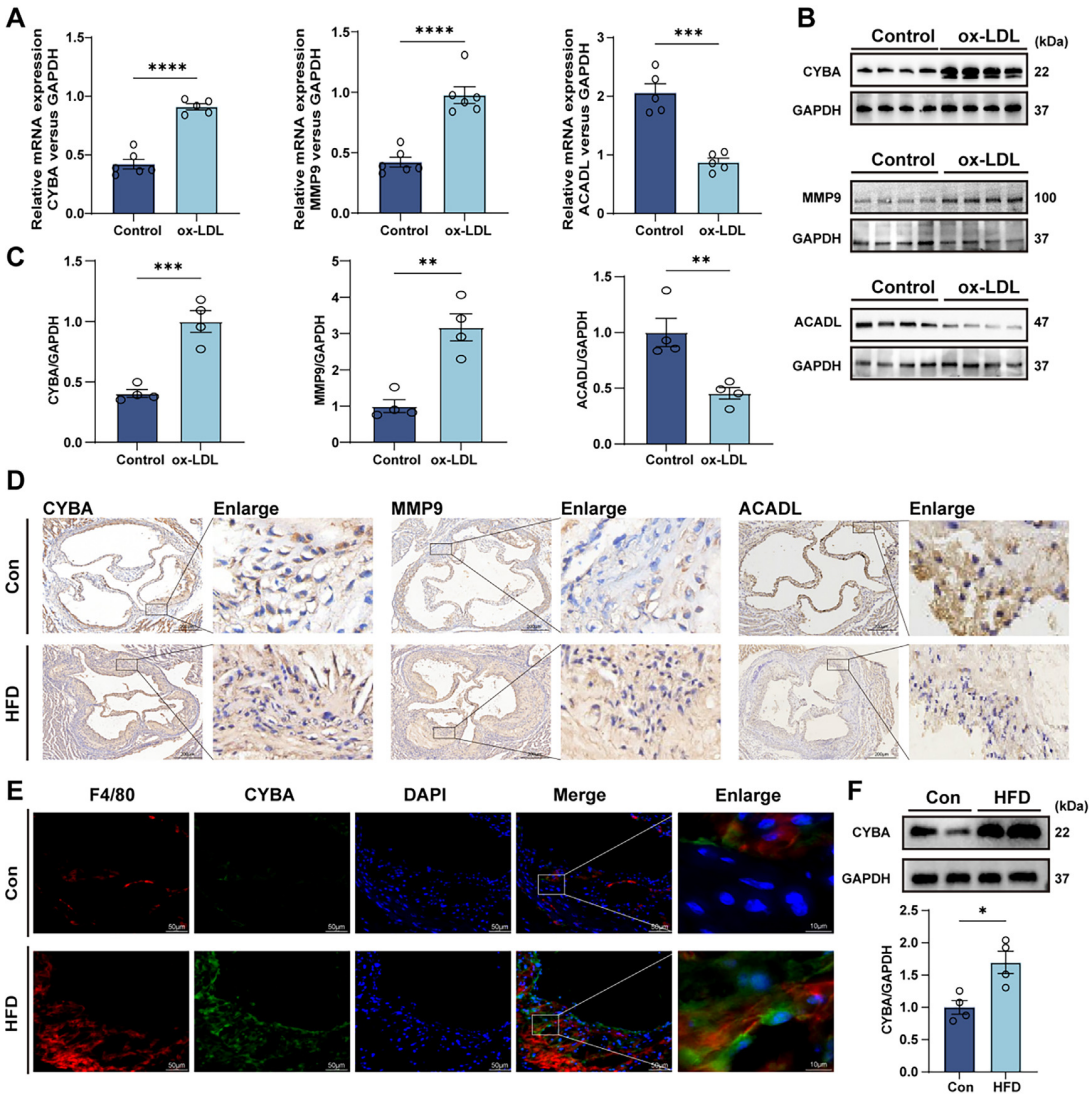
Validation of hub gene selection of CYBA for further study. (a) ox-LDL-induced upregulation of CYBA and MMP9 mRNA and downregulation of ACADL in THP-1 macrophages (n = 5–6). (b) and (c) Western blot analysis and quantification of hub genes expression in each group (n = 4). (d) Immunohistochemical images of hub genes with and without HFD (scale bar = 200 *μ*m). (e) Representative images of dual immunofluorescence staining of F4/80 and CYBA. Red fluorescence: F4/80; green fluorescence: CYBA; blue fluorescence: DAPI (scale bar = 50 *μ*m). (f) Western blot analysis demonstrated that HFD ApoE^−/−^ mice exhibited significantly upregulated CYBA protein expression in aortic tissue (n = 4). Data are presented as means ± SEM. ^*^p < 0.05; ^**^p < 0.01; ^***^p < 0.001; ^****^p < 0.0001.

To validate the expressions of five key genes, we utilized the THP-1 cell line—a widely adopted macrophage model in cardiovascular research.^22^ After stimulating the cells with ox-LDL for 24 h, we assessed the mRNA and protein levels of these genes. The results showed significant upregulation of CYBA and MMP9 [Figs. 6(a)–6(c)], aligning with our single-cell analysis, while ACADL was downregulated [Fig. 5(e)]. Neither REEP1 nor ADAP2 was detected in macrophages by RT-qPCR or WB (data not shown; see Supplementary Fig. S5 for IHC results). We further verified these findings in animal models. Hematoxylin and eosin (H&E) staining and Oil Red O staining demonstrated increased inflammatory cell infiltration and larger plaques in the HFD group (supplementary Fig. S4). Immunohistochemistry confirmed increased CYBA and MMP9 expression and decreased ACADL levels in HFD mice [Fig. 6(d)]. Prior research has established a correlation between CYBA and endothelial dysfunction in type 2 diabetes mellitus, as well as its association with subclinical atherosclerosis and vascular stiffness mediated by CYBA polymorphisms.^23,24^ These reports underscore the necessity for further exploration of CYBA's involvement in atherosclerosis. Additionally, dual immunofluorescence of mouse aortic sinus sections showed macrophage infiltration and colocalization of CYBA with F4/80^+^ macrophages—consistent with the findings from our bioinformatic analysis [Fig. 6(e)]. Subsequent WB analysis of aortic tissues also confirmed the upregulation of CYBA protein in HFD-fed mice [Fig. 6(f)].

### G. CYBA inhibition attenuates mitochondrial dysregulation and excessive ROS generation

**FIG. 7.**
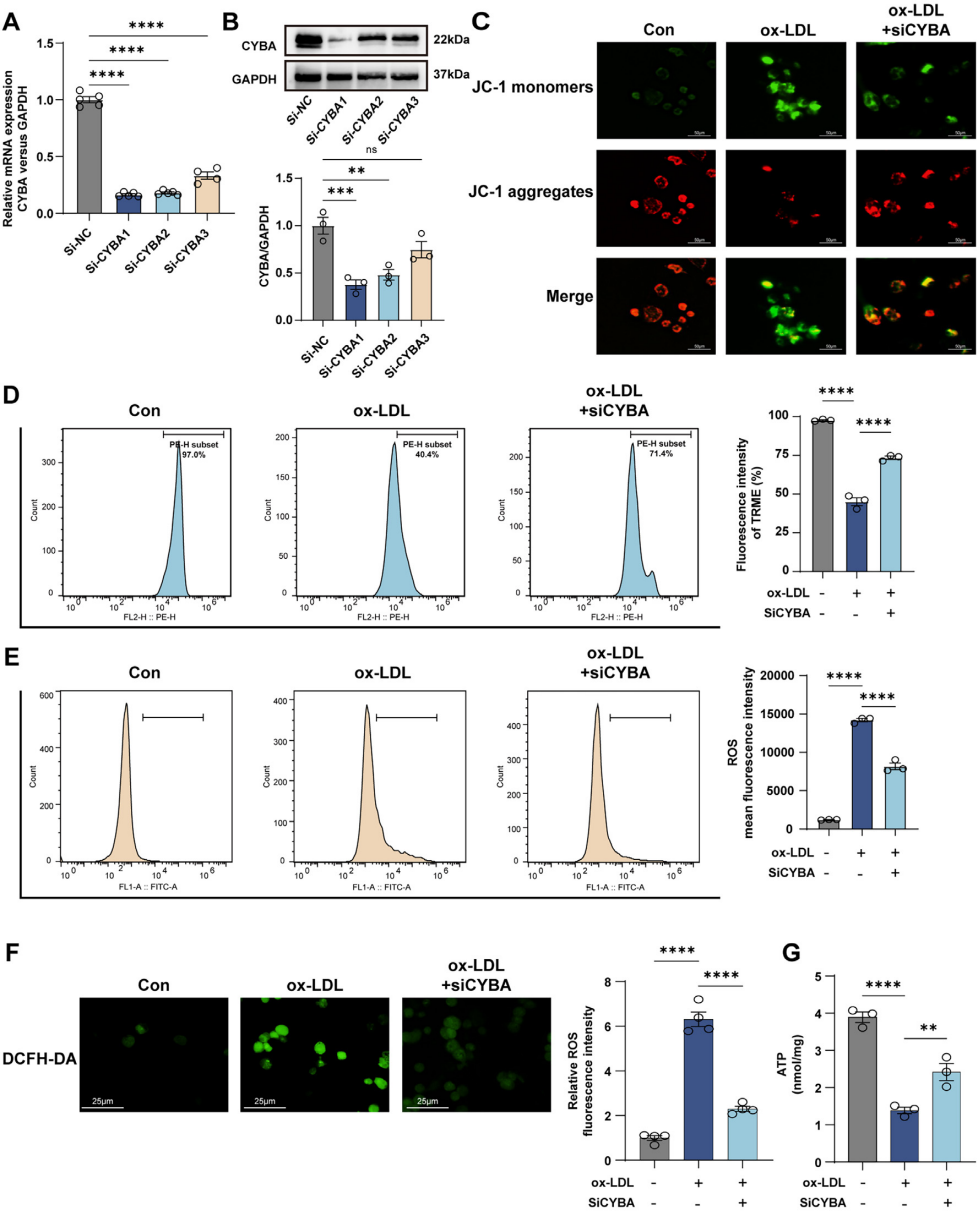
CYBA regulates mitochondrial activity and ROS production in THP-1 macrophages. (a) RT-qPCR analysis for the expression of CYBA in THP-1 transfected with siRNA (n = 4–5). (b) Western blot and quantification of CYBA protein levels in THP-1 transfected with siRNA and siCYBA1 exhibited the highest knockdown efficiency. siRNA was transfected at a final concentration of 50 nM (n = 3). (c) JC-1 fluorescence staining assessing the mitochondrial membrane potential in THP-1 cells. Red fluorescence: JC-1 aggregates; green fluorescence: JC-1 monomers (scale bar = 50 *μ*m). (d) TMRE-based flow cytometric quantification of mitochondrial membrane potential (n = 3). (e) Flow cytometry analysis demonstrated elevated intracellular ROS levels in THP-1 macrophages (n = 3). (f) ROS fluorescence detection images by DCFH-DA processing in different groups. Green fluorescence: ROS (scale bar = 25 *μ*m; n = 3). (g) Determination of the mitochondrial ATP levels in THP-1 macrophages (n = 3). Bars show the mean ± SEM. NS, not significant; ^**^p < 0.01; ^***^p < 0.001; ^****^p < 0.0001.

The CYBA gene encodes a crucial subunit of the NADPH oxidase complex. Given its biological association with oxidative respiratory activity (Fig. 4), we hypothesized that CYBA contributes to mitochondrial damage via regulation of ROS generation. To test this, we established a CYBA-knockdown model in THP-1 cells using small interfering RNA (siRNA), achieving a 62.2% reduction in protein expression [Figs. 7(a) and 7(b)]. Mitochondrial membrane potential was assessed using the JC-1 assay. Confocal microscopy showed that ox-LDL stimulation markedly increased JC-1 monomer formation (green fluorescence), which was reversed upon CYBA knockdown (enhanced red fluorescence aggregation) [Fig. 7(c)]. This result was further supported by TMRE-based flow cytometric quantification [Fig. 7(d)]. We next examined intracellular ROS levels using DCFH-DA fluorescence. Flow cytometry revealed that ox-LDL-induced ROS accumulation was significantly attenuated in si-CYBA-treated cells. Consistent with this, confocal imaging indicated a 5.3-fold increase in ROS fluorescence after ox-LDL treatment, which was effectively suppressed by CYBA knockdown [Figs. 7(e) and 7(f)]. Furthermore, the ox-LDL-induced depletion of ATP was also rescued following CYBA inhibition [Fig. 7(g)]. These results demonstrate that CYBA knockdown alleviates ox-LDL-induced mitochondrial impairment and aberrant ROS production.

### H. CYBA deficiency attenuates pro-inflammatory macrophage polarization and ferroptosis via ROS-dependent mechanisms *in vitro* and *in vivo*

**FIG. 8.**
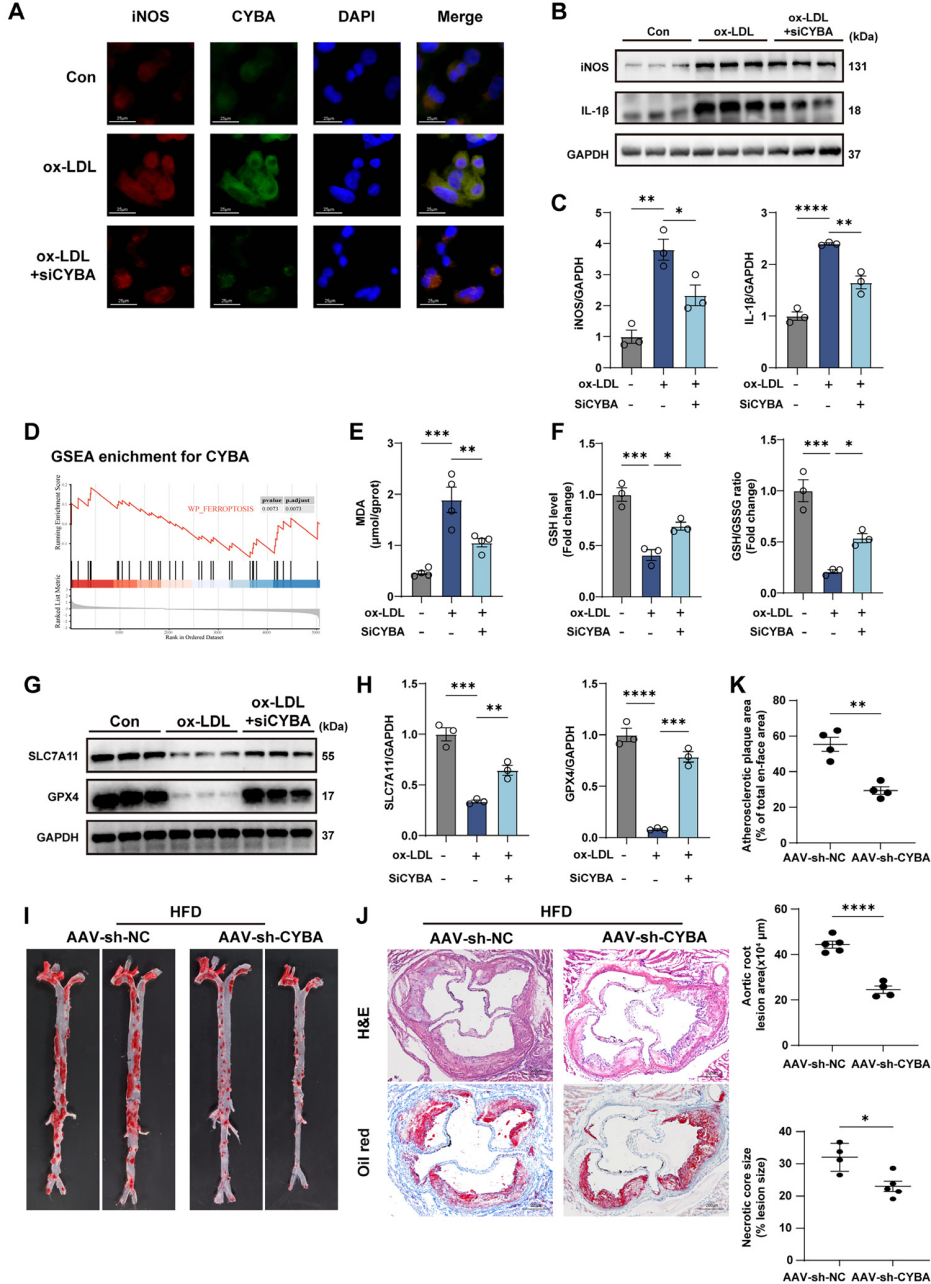

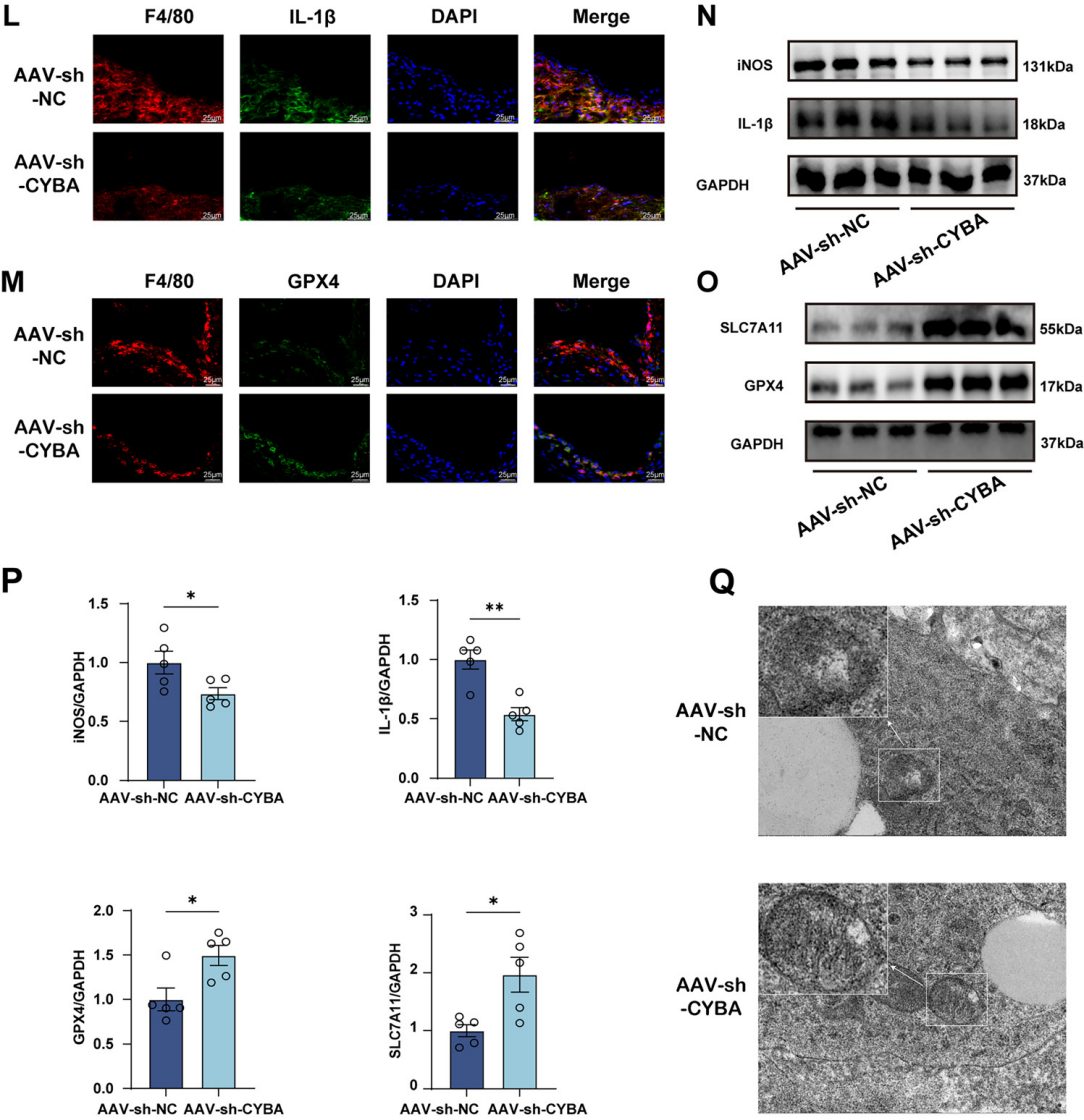
CYBA knockdown rescues inflammatory phenotype and ferroptosis in THP-1 macrophages and HFD mice. (a) Immunofluorescence co-staining of iNOS, CYBA. Red fluorescence: iNOS; green fluorescence: CYBA; blue fluorescence: DAPI (scale bar = 25 *μ*m). (b) and (c) Western blot analysis of iNOS and IL-1β expression in THP-1-derived macrophages (n = 3). (d) GSEA showed enrichment of the ferroptosis signaling pathway in macrophages. (e) Effect of knockdown of CYBA on MDA in macrophages (n = 4). (f) Relative levels of GSH and GSH/GSSG ratio in macrophages (n = 3). (g) Western blot quantification of ferroptosis regulators (GPX4, SLC7A11) in macrophages. (h) Quantification of ferroptosis-related proteins western blot results (n = 3). (i) Representative images of Oil Red O staining of whole-aorta en face Oil Red O staining. (j) Representative images of H&E and Oil Red O staining of the aortic sinus lesion area (scale bar = 200 *μ*m). (k) Quantitative analysis of plaque burden in the entire aorta (Oil Red O positive area/total en-face area), aortic sinus lesion area, and necrotic plaque content (necrotic core area/total plaque) based on Oil Red O and H&E staining (n = 4–5). (l) and (m) Representative images of dual immunofluorescence staining of F4/80, IL-1β/GPX4. Red fluorescence: F4/80; green fluorescence: IL-1β/GPX4; blue fluorescence: DAPI (scale bar = 25 *μ*m). (n)–(p) Western blot and quantification analysis of iNOS, IL-1β, GPX4, and SLC7A11 protein levels in aortic tissue (n = 5). (q) Representative transmission electron microscopy images of mitochondrial ultrastructure in aortic sinus plaques (magnification: ×12 000). Bars show the mean ± SEM. ^*^p < 0.05; ^**^p < 0.01; ^***^p < 0.001; ^****^p < 0.0001.

Emerging evidence establishes mitochondrial dysfunction as a key regulator of macrophage polarization and ferroptosis.^25,26^ Given our observation that CYBA knockdown preserves mitochondrial integrity and reduces ROS overproduction, we further explored its impact on these processes. Immunofluorescence [Fig. 8(a)] and WB analysis [Figs. 8(b) and 8(c)] revealed that ox-LDL stimulation promoted a pro-inflammatory macrophage phenotype in THP-1 macrophages, as evidenced by elevated expression of iNOS and IL-1β, which was attenuated by CYBA silencing. Re-analysis of single-cell transcriptomic data from macrophages stratified by CYBA expression indicated significant enrichment of ferroptosis-related pathways [Fig. 8(d)]. We next examined the effect of CYBA on lipid peroxidation, a hallmark of ferroptosis. Silencing CYBA suppressed ox-LDL-induced increases in MDA content [Fig. 8(e)], prevented the decline in GSH levels, and restored the GSH/GSSG ratio [Fig. 8(f)]. Furthermore, ox-LDL downregulated the expression of key anti-ferroptotic proteins GPX4 and SLC7A11, which was partially reversed by CYBA knockdown [Figs. 8(g) and 8(h)]. To evaluate the pathophysiological relevance of these findings, we employed an AAV-sh-CYBA-based knockdown approach in ApoE^−/−^ mice fed a HFD. WB analysis confirmed effective CYBA knockdown in aortic tissue (supplementary Fig. S6). CYBA deficiency significantly reduced atherosclerotic plaque area by 26.1% [Figs. 8(i)–8(k)]. Consistent with *in vitro* results, CYBA knockdown suppressed pro-inflammatory mediators and restored ferroptosis defense mechanisms *in vivo* [Figs. 8(l)–8(p)]. We also observed that CYBA knockdown *in vivo* ameliorated mitochondrial damage within aortic sinus plaques of HFD-fed mice: mitochondria in the HFD group exhibited vacuolation and dissolution of double membranes, whereas those in sh-CYBA-treated mice showed markedly less structural damage [Fig. 8(q)]. In conclusion, CYBA deficiency attenuates pro-inflammatory polarization and ferroptosis, suggesting that CYBA promotes these processes via ROS-induced mitochondrial dysfunction.

### I. CYBA regulates macrophage polarization and ferroptosis via the Nrf2/HO-1/NQO-1 axis

**FIG. 9.**
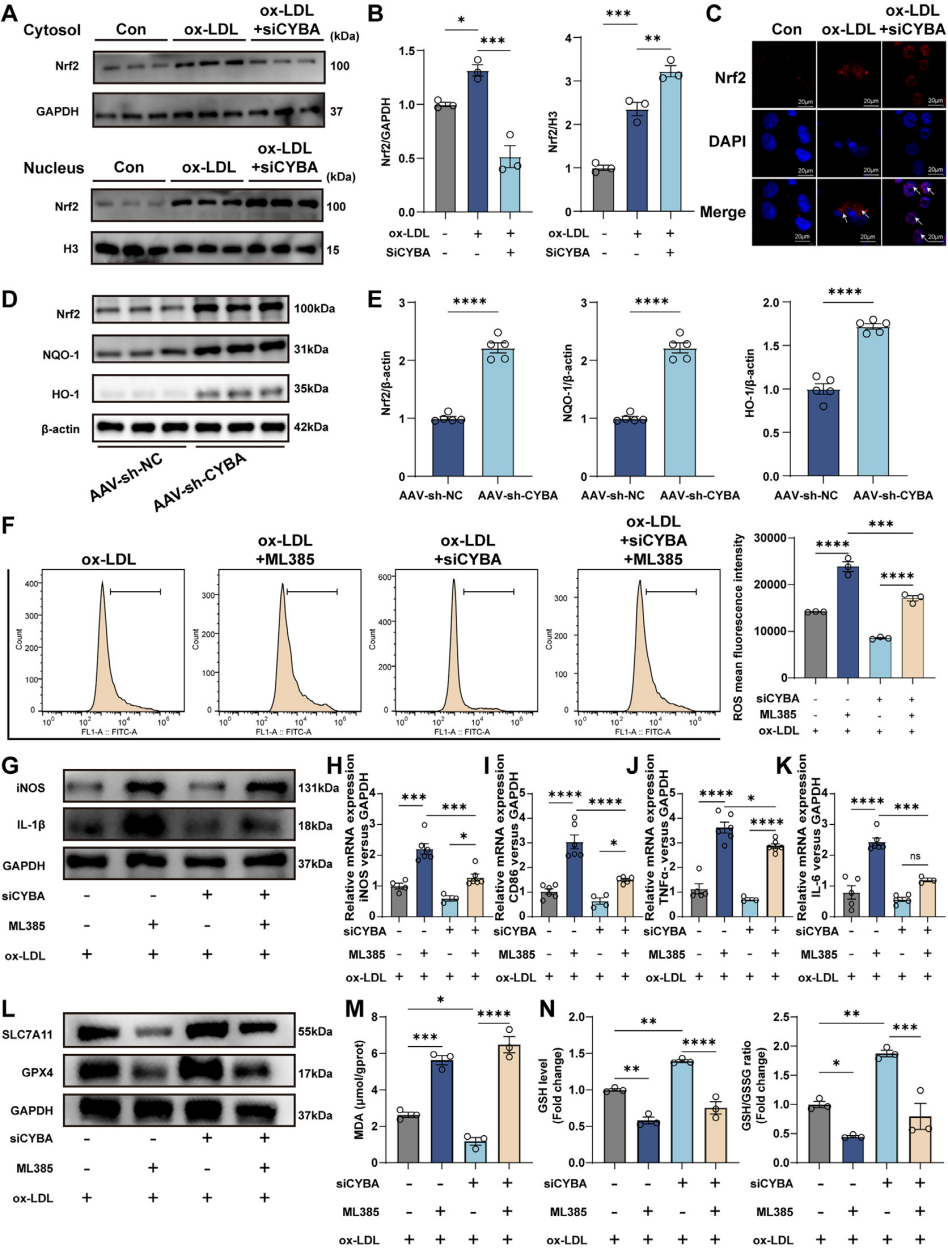
CYBA regulates macrophage polarization and ferroptosis through the Nrf2/HO-1/NQO-1 pathway. (a) and (b) Western blot illustrated the accumulation of Nrf2 in the nucleus after knockdown of CYBA in THP-1 macrophages (n = 3). (c) Representative immunofluorescence images depicting the localization of Nrf2 in the cytoplasm and nucleus. The white arrows indicate regions where Nrf2 is localized in the cytoplasm and nucleus (scale bar = 20 *μ*m). (d) and (e) Nrf2 pathway activation in aortic tissues from 12-week HFD mice (n = 5). (f) Intracellular ROS levels were quantified by flow cytometry (n = 3). (g) Representative protein expression of pro-inflammatory markers (iNOS, IL-1β) in THP-1 macrophages. (h)–(k) mRNA expression levels of iNOS, CD86, TNF-α, and IL-6 across experimental groups (n = 3–6). (l) Representative Western blot analysis of SLC7A11 and GPX4 in THP-1 macrophages. (m) The levels of MDA in THP-1 macrophages were determined using commercial kits (n = 3). (n) Relative levels of GSH and GSH/GSSG ratio in THP-1 macrophages (n = 3). Bars show the mean ± SEM. NS, not significant; ^*^p < 0.05; ^**^p < 0.01; ^***^p < 0.001; ^****^p < 0.0001.

We next investigated whether the Nrf2/HO-1/NQO-1 pathway, a central antioxidant signaling axis critical for mitochondrial homeostasis,^27^ mediates the effects of CYBA on macrophage polarization and ferroptosis. We first examined the activation of the Nrf2 pathway following CYBA knockdown. WB analysis showed increased nuclear translocation of Nrf2 upon siCYBA treatment [Figs. 9(a) and 9(b)], which was further confirmed by immunofluorescence staining [Fig. 9(c)]. Consistently, aortic tissues from HFD-fed AAV-sh-CYBA mice exhibited enhanced activation of the Nrf2/HO-1/NQO-1 pathway [Figs. 9(d) and 9(e)]. To determine whether Nrf2 activation mediates the protective effects of CYBA knockdown, we used the Nrf2 inhibitor ML385. Nrf2 inhibition abolished the reduction in ROS levels induced by CYBA silencing [Fig. 9(f)], reversed the suppression of pro-inflammatory polarization—evidenced by elevated iNOS, CD86, and TNF-α [Figs. 9(g)–9(k)]—and attenuated the anti-ferroptotic effects, reducing GPX4 and SLC7A11 expression [Fig. 9(l)], increasing MDA content [Fig. 9(m)], and decreasing GSH levels and GSH/GSSG ratio [Fig. 9(n)]. Collectively, these results demonstrate that CYBA represses Nrf2 nuclear translocation and downstream antioxidant responses. CYBA deficiency activates the Nrf2/HO-1/NQO-1 pathway, ultimately attenuates oxidative stress, pro-inflammatory polarization, and ferroptosis.

## III. DISCUSSION

**FIG. 10.**
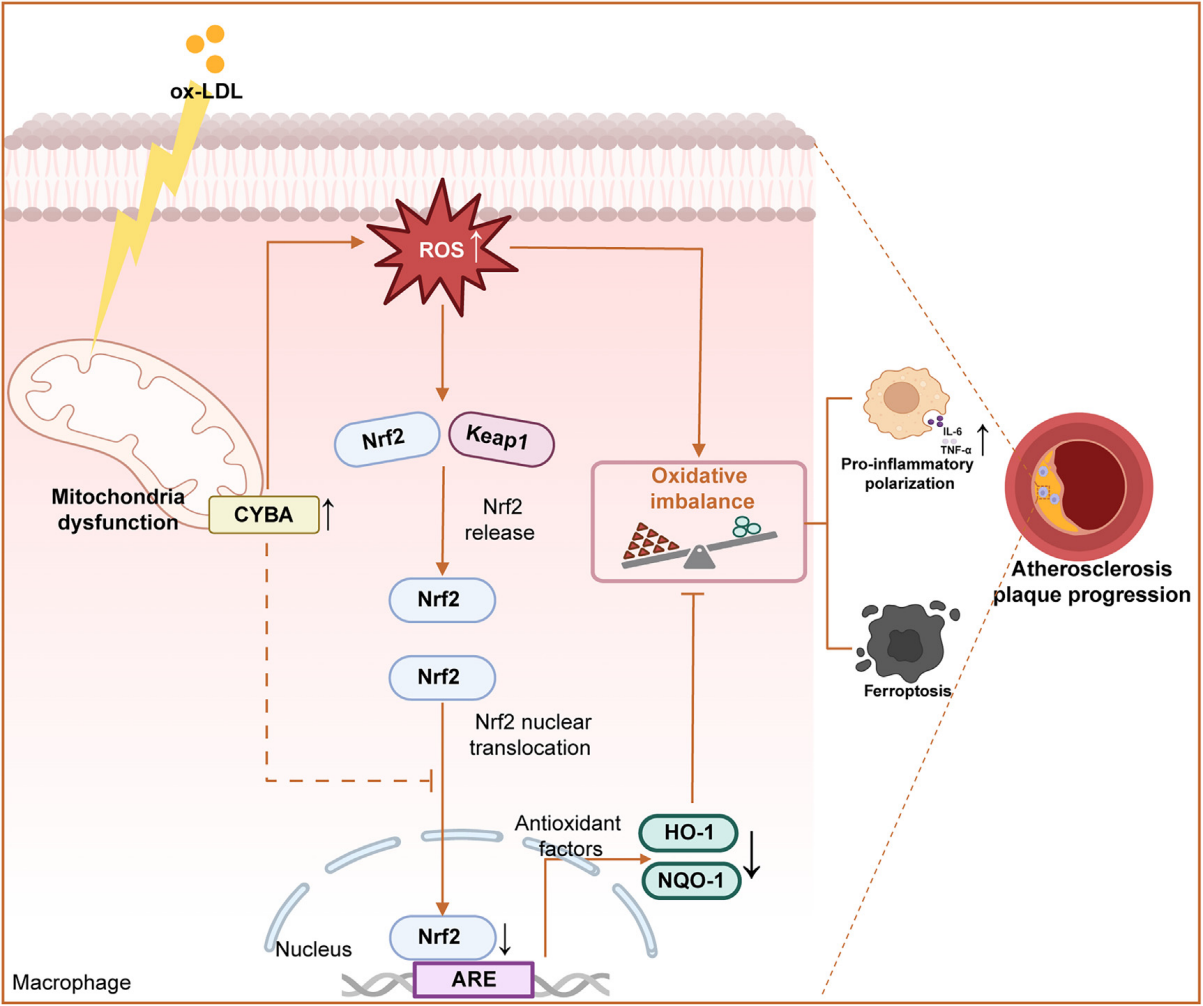
Working model for CYBA-mediated macrophage dysregulation in atherosclerosis.

Atherosclerotic plaque progression is fundamentally driven by mitochondrial dysfunction in cholesterol-laden macrophages,^28^ yet the precise mechanisms linking mitochondrial damage to immune dysregulation remain elusive. Our study uncovers that CYBA overexpression in atherosclerotic macrophages disrupts the canonical ROS-Nrf2 antioxidant axis. Instead of inducing compensatory Nrf2/HO-1/NQO-1 activation in response to mitochondrial-derived ROS, CYBA initiates a self-amplifying oxidative cascade by aggravating mitochondrial electron transport chain defects to escalate ROS generation while concurrently suppressing Nrf2 nuclear translocation to inhibit antioxidant defenses. This dual impairment creates a redox imbalance that promotes pro-inflammatory macrophage polarization and triggers ferroptosis, accelerating atherosclerotic plaque progression (Fig. 10).

Our immune landscape analysis revealed a significant reduction in circulating monocytes alongside pronounced infiltration of macrophage populations, particularly M1 subtypes, within atherosclerotic lesions. This cellular shift suggests accelerated monocyte-to-macrophage differentiation coupled with microenvironment-driven polarization.^19,29^ Macrophages infiltrating the plaques play a central role in lipid metabolism and inflammatory responses, where ox-LDL promotes their polarization toward an M1 phenotype that secretes pro-inflammatory cytokines, thereby exacerbating local inflammation and promoting AS progression. Mitochondrial dysfunction further amplifies oxidative stress, inflammatory activation, and cell death, playing a critical role in the initiation and development of atherosclerosis.^30^ Here, our multi-omics integration establishes CYBA as a molecular linchpin bridging mitochondrial dysfunction to phenotypic reprogramming and ferroptotic commitment. Although CYBA polymorphisms have been epidemiologically linked to cardiovascular risk,^31^ its mechanistic contribution to atherogenesis remains enigmatic. We provide the first experimental evidence that CYBA orchestrates Nrf2 dysregulation, thereby driving atherosclerotic progression. Mechanistically, while CYBA physiologically helps maintain intracellular redox homeostasis, its pathological overexpression in atherosclerosis disrupts the mitochondrial-immune crosstalk. Functional validation confirmed that CYBA knockdown restored mitochondrial membrane potential, attenuated lipid peroxidation, rescued ATP production, and suppressed M1-like polarization. Crucially, we uncovered that CYBA impedes Nrf2 nuclear translocation, trapping it in the cytoplasm.

The atheroprotection observed upon systemic CYBA knockdown likely reflects combined effects across multiple plaque cell types. Our scRNA-seq data [Fig. 5(e)] confirms CYBA expression in both ECs and VSMCs. Emerging evidence suggests that CYBA/NOX-derived ROS critically contribute to EC dysfunction by promoting oxidative stress,^32^ which can impair endothelial barrier integrity and upregulate adhesion molecules, thereby facilitating monocyte recruitment. In VSMCs, CYBA-dependent ROS generation is implicated in driving phenotypic switching from a contractile to a synthetic state,^33^ stimulating excessive proliferation and migration, processes central to pathological vascular remodeling and fibrous cap stability. The ongoing development of CYBA-targeted inhibitors for sterile inflammatory diseases—exemplified by their efficacy in suppressing NLRP3 inflammasome activation and IL-1β/IL-18 secretion in rheumatoid arthritis^34,35^—further highlights the translational promise of modulating this axis in atherosclerosis, another condition fundamentally driven by chronic sterile inflammation. Thus, systemic CYBA knockdown likely confers atheroprotection not only through macrophage reprogramming but also by cultivating a globally anti-inflammatory plaque microenvironment. Collectively, these findings establish CYBA as a promising therapeutic target for mitigating atherosclerosis. Notably, CYBA may also signal through non-Nrf2 pathways. The partial rescue of inflammation upon Nrf2 inhibition suggests complementary mechanisms. Our GSEA analysis shows that CYBA^high^ macrophages exhibit activation of pro-inflammatory pathways such as IL-6/JAK/STAT3 and TNF-α/NF-κB [Fig. 4(c)]. Studies have confirmed that ROS can directly activate both the JAK/STAT and NF-κB pathways, thereby initiating and amplifying inflammatory responses.^36,37^ Furthermore, sustained activation of STAT3 can suppress mitochondrial function and induce aerobic glycolysis, establishing a link between metabolic reprogramming and inflammation.^38^ Upstream transcriptional regulation may also contribute to this process; for instance, the transcription factor PU.1 can directly promote CYBA expression, which in turn exacerbates mitochondrial oxidative stress.^39^ Therefore, we speculate that within the atherosclerotic milieu, CYBA may be upregulated by factors like PU.1, while also activating other parallel, non-Nrf2-dependent signaling cascades. These mechanisms likely cooperate to shape the robust inflammatory phenotype of macrophages, a premise that warrants further investigation in future studies.

ACADL is crucial for mitochondrial fatty acid oxidation, facilitating the initial steps of this metabolic pathway.^40^ Although direct evidence linking ACADL to atherosclerosis is lacking, its association with metabolic disorders and malignancies has been documented.^41,42^ Deficiencies or losses of ACADL impair β-oxidation of fatty acids, reducing macrophage accumulation and subsequently activating peroxisome proliferator-activated receptor (PPAR). PPAR, by antagonizing the NF-κB signaling pathway, exhibits anti-inflammatory properties.^43^ Research has demonstrated that lipopolysaccharide (LPS) can worsen mitochondrial long-chain fatty acid β-oxidation to aggravate alveolar inflammation.^44^ Wang *et al.*^45^ demonstrated that ACADL overexpression boosts fatty acid oxidation in the myocardium, thereby reducing hypertrophy and enhancing function. Despite observed downregulation of ACADL in AS lesions, our GSEA enrichment analysis suggests that its elevated expression correlates with anti-inflammatory effects, implying a compensatory feedback mechanism warranting further study.

MMP9, mainly produced by macrophages, is closely linked to plaque instability and rupture, with low expression in normal tissues. It degrades the extracellular matrix and blood vessel basement membrane, weakening the fibrous cap and promoting rupture.^46^ During the progression of atherosclerosis, inflammatory factors such as LPS, ox-LDL, and mitochondrial ROS stimulate MMP9 production in macrophages.^47,48^ Simultaneously, MMP9 can induce macrophage polarization toward the pro-inflammatory M1-like phenotype, thereby amplifying the inflammatory response. This creates a feedback loop that worsens plaque instability. Research by De Paoli *et al.*^49^ has shown that enhancing the expression of neuron-derived orphan receptor 1 on macrophage membranes can inhibit MMP expression, thereby promoting macrophage polarization toward the anti-inflammatory M2-like phenotype. To summarize, MMP9 is a promising therapeutic target for AS treatment.

While our study delineates CYBA's central role in mitochondrial-immune crosstalk, there were also some limitations that warrant attention: (1) We unveiled the induction of mitochondrial damage in the context of macrophage CYBA knockdown, but the precise molecular mechanisms linking CYBA-mediated mitochondrial damage to M1-like polarization/ferroptosis require deeper exploration. (2) Although we established Nrf2's involvement, the molecular determinants of CYBA-Nrf2 interaction (e.g., post-translational modifications) remain undefined, future research should aim to elucidate the specific mechanisms by which CYBA regulates Nrf2 pathway. (3) Given CYBA's ubiquitous expression, its contributions to other plaque cells (e.g., endothelial dysfunction, smooth muscle cell senescence) and intercellular crosstalk mechanisms demand further exploration.

## IV. CONCLUSION

In conclusion, our findings establish CYBA as a critical therapeutic target in atherosclerosis; ox-LDL-induced upregulation of CYBA in macrophages drives excessive ROS production, leading to redox imbalance and mitochondrial damage. Concurrently, CYBA impairs the Nrf2/HO-1/NQO-1 cytoprotective pathway, crippling antioxidant responses. These effects collectively promote pro-inflammatory macrophage polarization and ferroptosis, ultimately accelerating plaque progression. Therapeutic inhibition of CYBA represents a promising strategy to restore redox homeostasis and mitigate atherosclerosis development.

## V. METHODS

### A. Public data acquisition and preparation

The datasets utilized in this research were sourced from the Gene Expression Omnibus (GEO) database (http://www.ncbi.nlm.nih.gov/geo). A total of seven databases were acquired, as specified in Supplementary Table S1. Samples were procured from datasets GSE28829 and GSE226790, comprising 19 AS samples and 16 control samples, which served as the training set for the identification of differentially expressed genes (DEGs) using the “limma” package in R. To mitigate batch effects and enhance the reliability of the analytical outcomes, the “sva” package in R was employed. For a more intuitive presentation and interpretation of the data, “ggplot2” and “pheatmap” were utilized to generate volcano plots and heatmaps, respectively. Additionally, we obtained 2030 human mitochondrial-related genes from the MitoCarta3.0 database (http://www.broadinstitute.org/mitocarta) and the Gene Set Enrichment Analysis (GSEA) database (http://www.gseamsigdb.org/gsea/msigdb/index.jsp).^50,51^ The Venn diagram was created using the “Venn” package in R software.

### B. WGCNA-based module construction and trait correlation study

In this study, the Weighted Gene Co-expression Network Analysis (WGCNA) was conducted utilizing the “WGCNA” package.^52^ Initially, a sample dendrogram was constructed using hierarchical clustering to filter the samples. Subsequently, an appropriate soft threshold was determined by examining the relationship between the power parameter (β) and both the Scale-Free Topology Model Fit (signed R^2^) and Mean Connectivity. Following this, the Topological Overlap Matrix (TOM) was computed by applying the selected soft threshold. Gene ratios and associated differences were then calculated. To visualize the correlation between modules and traits, scatter plots were employed to illustrate the relationship between Module Membership (MM) and Gene Significance (GS) within key modules. In summary, WGCNA allows for the association of modules with external traits, helping us identify potential biological markers.

### C. Exploration of immune characteristics

We employed the “CIBERSORT” analytical package to quantify the relative abundance of various immune cell types within the samples. CIBERSORT utilizes established gene expression profiles for 22 distinct immune cell types to develop a linear model. In particular, we applied the LM22 gene expression signature matrix, which encompasses the characteristic gene expression data for these 22 immune cell types. This approach enabled us to evaluate immune cell infiltration levels in the AS group relative to the control group.

### D. Identification of biomarkers through multiple machine learning techniques

We employed a comprehensive approach by utilizing 12 distinct algorithms for variable selection and predictive modeling, including Lasso, Ridge, Enet, Stepglm, SVM, glmBoost, LDA, plsRglm, RandomForest, GBM, XGBoost, and NaiveBayes, culminating in a total of 113 model combinations. To enhance the sample size and improve the precision of our selection process, we re-extracted data from GSE28829, GSE43292, GSE57691, GSE100927, and GSE21545 datasets and integrated the data using the “sva” package, resulting in a cohort comprising 252 cases of AS and 90 controls. Subsequently, we partitioned the data into training and testing sets with a 7:3 ratio, with the training cohorts consisting of 176 AS samples and 62 control samples, and the testing cohorts containing 76 AS samples and 28 control samples. Next, we proceeded to calculate the area under the curve (AUC) for these models across both cohorts and visualized the model evaluation outcomes through a heatmap. For the Random Forest (RF) algorithm, the implementation utilizes the “randomForest” package in R.

### E. Nomogram development and ROC curve evaluation for biomarker validation

To assess the diagnostic efficacy of the selected genes, we examined their capacity to differentiate between AS and control cohorts within both the training and validation datasets. The diagnostic performance was visualized using the area under the curve (AUC) generated by the “pROC” R package, with higher AUC values signifying enhanced predictive power of the key genes. Furthermore, we employed the “rms” R package to construct a nomogram, which represents the predictive outcomes derived from the logistic regression model.

### F. Functional enrichment analysis

Gene Ontology (GO) and Kyoto Encyclopedia of Genes and Genomes (KEGG) enrichment analyses were performed utilizing the “clusterProfiler” R package. These analyses are designed to identify biological processes, molecular functions, and cellular components that are overrepresented within a specified set of genes, thereby providing insights into the functional implications of gene expression data. In this study, p-values below 0.05 were deemed statistically significant. Additionally, Gene Set Enrichment Analysis (GSEA) was conducted using the hallmark gene set as a reference framework. This method facilitates the evaluation of whether predefined gene sets demonstrate statistically significant differences in expression between two biological states. Pathways were considered enriched when the p-value was less than 0.05.

### G. Protein–protein interaction, transcription factor, and miRNA network analysis

A protein–protein interaction (PPI) network and co-expression interactions for common genes were constructed using the GeneMANIA website^53^ (genemania.org) to elucidate the molecular interactions and regulatory mechanisms. Transcription factors (TFs) were predicted using multiple databases, including GTRD (http://gtrd.biouml.org/), KNOCK-TF (www.licpathway.net/KnockTF/), and the ChIP-Atlas (http://chip-atlas.org).^54–56^ To enhance the accuracy of TF predictions, the results from these databases were integrated. Additionally, the TargetScan (http://www.targetscan.org) tool was employed to predict miRNAs, and the results were visualized through Cytoscape software.^57^

### H. Single-cell RNA sequencing analysis

Single-cell RNA sequencing data derived from human atherosclerotic core plaques were acquired from the GEO repository (accession GSE159677), consisting of three fully calcified atherosclerotic lesions and three patient-matched proximal adjacent control tissues.^58^ Raw data processing was performed using the Seurat R package (version 4.0.2) with rigorous quality control (QC) procedures. Specifically, cells meeting the following thresholds were retained: (1) 200–4500 expressed genes per cell and (2) mitochondrial gene content <20% of total transcripts. Subsequent data processing included log-normalization and scaling through a linear regression framework employing log-transformed normalization. The top 2000 highly variable genes were identified using the “FindVariableFeatures” algorithm implementing variance-stabilizing transformation. Dimensionality reduction was performed through principal component analysis (PCA) with significant principal components selected by the elbow method. Cell clusters were visualized using uniform manifold approximation and projection (UMAP). Cell type annotation was validated through canonical marker gene expression patterns: T cells (CD4), vascular smooth muscle cells (ACTA2, MYH11), endothelial cells (ECSCR, VWF), macrophages (CD14, CD68), NK_T cells (NKG7, TRDC), B cells (CD19, MS4A1), plasmacytoid dendritic cells (CLEC4C, IRF7), and mast cells (TPSAB1).

### I. Cell culture and siRNA transfection

THP-1 cells were procured from Procell Life Science & Technology Co. Ltd. and maintained in RPMI 1640 medium supplemented with 10% fetal bovine serum (FBS). The cells were incubated at 37 °C in a humidified atmosphere with 5% CO_2_ and 95% air. Initially, THP-1 cells were induced with 100 nM phorbol 12-myristate 13-acetate (PMA) (MCE, NJ, USA) for 48 h to facilitate adhesion and polarization into M0 macrophages. For Nrf2 inhibition, ML385 was administered for 24 h before subsequent treatments. Following these pretreatments, the cells were exposed to 100 *μ*g/ml ox-LDL from Yiyuan Biotechnology, Guangzhou, China, for an additional 24 h.

Transfection of the cells was performed using 50 nM CYBA-specific small interfering RNA (siRNA) oligonucleotides (siCYBA) or negative control siRNA oligonucleotides (siNC) with the CALNP™ RNAi *in vitro* reagent (Beijing, China) for 24 h to assess mRNA expression and for 48 h to evaluate protein expression, following the manufacturer's protocol.

### J. Animal model

All 24 male ApoE^−/−^ mice (6–8 weeks old) were purchased from Biont Biological Technology Co., Ltd. (Wuhan, China). The mice were housed under specific pathogen-free conditions at the Animal Center of Tongji Medical College with free access to food and water. After a 1-week acclimation period, the mice were randomly divided into groups and subjected to different experimental conditions. To induce the atherosclerosis model, the mice were fed a high-fat/high-cholesterol diet (HFD; comprising 20% fat and 1.25% cholesterol; D12108C, Medicience, Ltd.) for 12 weeks. To achieve CYBA knockdown in ApoE^−/−^ mice, adeno-associated virus (AAV) vectors carrying short hairpin RNA (shRNA) were designed and synthesized by Shanghai Genechem (Shanghai, China). In the AAV-sh-NC group, ApoE^−/−^ mice received a single intravenous injection of 1.0 × 10^12^ plaque-forming units of scrambled shRNA (100 *μ*l/mouse, sequence: CGCTTCCGCGGCCCGTTCAA). Similarly, for the AAV-sh-CYBA group, mice received 1.0 × 10^12^ plaque-forming units of AAV via intravenous injection (sequence: CGTTTCACACAGTGGTATTTC). After a 2-week acclimatization, mice were placed on the high-fat/high-cholesterol diet. At study termination, all mice were euthanized. Blood was collected from the retro-orbital plexus, and serum was separated by centrifugation (3000 rpm, 15 min, 4 °C) and stored at −80 °C for subsequent biochemical analysis. The levels of total cholesterol, LDL, HDL, and triglycerides were measured using commercial assay kits (Nanjing Jiancheng Biological Co., Ltd.) according to the manufacturer's instructions. Subsequently, mice were perfused with saline via the left ventricle. The entire aortic tree from the aortic root to iliac bifurcation was meticulously dissected, cleared of perivascular adipose tissue, and either snap-frozen in liquid nitrogen or fixed in 4% paraformaldehyde for subsequent analyses. All experimental procedures involving the mice adhered to the guidelines set forth in the National Institutes of Health Guide for the Care and Use of Laboratory Animals. Approval for all animal procedures was obtained from the Institutional Animal Care and Use Committee (IACUC) of Huazhong University of Science and Technology (IACUC number [3628]).

### K. Quantitative real-time PCR analysis

Total RNA from cells was extracted utilizing the RNA-easy Isolation Reagent (Cat. No. R701-02, Vazyme, China). Following RNA extraction, quantification was performed using a NanoDrop spectrophotometer, with the A260/A280 ratio measured to assess RNA purity. Complementary DNA was synthesized from 2 *μ*g of total RNA in a 20 *μ*l reaction volume using the HiScript^®^ III RT SuperMix for qPCR (Cat. No. R323-01, Vazyme, China), with the reaction conducted at 42 °C and subsequently inactivated at 95 °C. Real-time quantitative PCR was then carried out using the ChamQ SYBR qPCR Master Mix (Cat. No. Q311-02, Vazyme, China) on a CFX96 real-time PCR system (Bio-Rad, CA, USA), following the manufacturer's protocol. The protocol included an initial incubation at 96 °C for 15 min, followed by cycles of 45 s at 96 °C, 30 s at the annealing temperature, and 10 s at 72 °C. All procedures were conducted using filter tips and RNase-free tubes to prevent contamination. Target mRNA levels were normalized to GAPDH mRNA levels, and data analysis was performed using the 2^−ΔΔCt^ method. Primer sequences are provided in Supplementary Table S2.

### L. Western blot

Total protein was extracted from cells lysed with RIPA buffer (Servicebio, G2002, Wuhan, China), supplemented with phosphatase inhibitors (Servicebio, G2007, Wuhan, China), to enhance protein yield. Nuclear protein was isolated using a nuclear protein extraction kit (Beyotime, P0027, Shanghai, China). The protein concentration of the samples was quantified using the BCA protein assay kit (Thermo Scientific, 23225, MA, USA). Subsequently, the extracted proteins were separated by sodium dodecyl sulfate–polyacrylamide gel electrophoresis (SDS–PAGE) and transferred onto polyvinylidene fluoride (PVDF) membranes. Following a blocking procedure with protein-free rapid blocking buffer (EpiZyme, PS108P, Shanghai, China) for 30 min, the membranes were incubated with specific primary antibodies targeting the proteins of interest overnight at 4 °C. The primary antibodies employed included CYBA (1:1000, ABclonal, Wuhan, China), ACADL (1:1000, ABclonal, Wuhan, China), MMP9 (1:1000, ABclonal, Wuhan, China), ADAP2 (1:500, Sanying, Wuhan, China), REEP1 (1:1000, Sanying, Wuhan, China), GAPDH (1:50 000, Sanying, Wuhan, China), β-actin (1:20 000, Sanying, Wuhan, China), iNOS (1:1000, Abcam, USA), IL-1β (1:1000, Abcam, USA), H3 (1:2000, Sanying, Wuhan, China), Nrf2 (1:2000, ABclonal, Wuhan, China), HO-1 (1:2000, Sanying, Wuhan, China), NQO-1 (1:5000, Sanying, Wuhan, China), GPX4 (1:1000, ABclonal, Wuhan, China), and SLC7A11 (1:1000, ABclonal, Wuhan, China). Following this, the membranes were then washed and incubated with 1:10 000 HRP-conjugated secondary antibodies, goat anti-rabbit, and goat anti-mouse, respectively. Detection of protein bands was performed using horseradish peroxidase-conjugated secondary antibodies and an enhanced chemiluminescence reagent, with visualization achieved through the application of a chemiluminescence reagent (Bio-Rad, CA, USA).

### M. Histology analysis

To evaluate the lesion size, the entire aortic vessel tree, extending from the ascending aorta to the bifurcation of the common iliac arteries, was isolated and stained with Oil Red O. The aortic root was embedded in optimal cutting temperature compound (OCT) medium, cryosectioned into 5 *μ*m slices, and subjected to either immunofluorescence or immunohistochemical analysis. The sections underwent staining with hematoxylin and eosin (H&E) and Oil Red O, as well as incubation with primary antibodies, including CYBA (1:100, ABclonal, Wuhan, China), ACADL (1:100, ABclonal, Wuhan, China), MMP9 (1:50, ABclonal, Wuhan, China), ADAP2 (1:100, Sanying, Wuhan, China), and REEP1 (1:100, Sanying, Wuhan, China). Immunofluorescence staining was conducted in accordance with the manufacturer's protocol to detect F4/80 (1:50, Abcam, USA) and CYBA (1:100, ABclonal, Wuhan, China). Images were acquired using an Olympus VS200 slideview microscope.

### N. Immunofluorescence staining

THP-1 cells were fixed in 4% paraformaldehyde at room temperature for 30 min and subsequently washed three times with phosphate-buffered saline (PBS). In brief, the specimens were permeabilized using 1% Triton X-100 and blocked with a solution containing 1% bovine serum albumin (BSA) and 3% fetal bovine serum (FBS) in PBS. The cells were then stained with iNOS at a dilution of 1:500 (Abcam, USA) and CYBA at a dilution of 1:50 (ABclonal, Wuhan, China) overnight at 4 °C. Following this, the cells were incubated with secondary antibodies at room temperature for 1 h. Nuclear staining was performed using 4′,6-diamidino-2-phenylindole (DAPI). Imaging was conducted using a confocal microscope (Olympus FV1200 SPECTRAL Laser Scanning Confocal Microscope).

### O. ROS detection

THP-1-derived macrophages were seeded in 6-well plates and polarized into the M0 phenotype with 100 nM PMA. Following experimental treatments (ox-LDL and/or ML385 exposure for 24 hr according to experimental design), cells were incubated with 10 *μ*M DCFH-DA (dissolved in serum-free medium) at 37 °C under light-protected conditions for 20 min. After removal of the staining solution, cells were gently washed three times with pre-warmed serum-free medium to eliminate extracellular DCFH-DA residuals. For fluorescence microscopy, images were captured immediately using a fluorescence microscope. For flow cytometric quantification, cells were detached using non-enzymatic cell dissociation buffer, centrifuged at 300 × g for 5 min, and resuspended in PBS. Fluorescence intensity was measured on a BD LSRFortessa™ flow cytometer (BD Biosciences, USA). Data analysis was performed using FlowJo V10 software, with ROS levels expressed as geometric mean fluorescence intensity (MFI) normalized to untreated controls.

### P. Transmission electron microscope (TEM) of aortic tissues

Fresh mouse aortic plaque samples were dissected into small pieces (approximately 1 mm^3^) and fixed in electron microscopy fixative. Briefly, after collection, the tissues underwent fixation, dehydration, embedding, polymerization, and staining steps according to standard TEM protocols. Ultrastructural examination was performed using a Hitachi transmission electron microscope (Hitachi, HT7800, Japan), and representative images were acquired for analysis.

### Q. Reagents

The ROS levels were measured using the ROS Detection Kit (Cat. No. S0033S), and mitochondrial membrane potential was assessed with the JC-1 (Cat. No. C2006) and TRME (Cat. No. C2001S) kits. The levels of reduced and oxidized glutathione (GSH and GSSG) were determined employing the GSH and GSSG assay kits (Cat. No. S0053). Additionally, MDA content in cells was determined using a specific kit (Cat. No. S0131S), while ATP levels in cell lysates were measured with the ATP Assay Kit (Cat. No. S0026). All aforementioned kits were purchased from Beyotime, Shanghai, China, and the experiments were performed according to the manufacturer's instructions. The Nrf2-specific inhibitor ML385 (Cat. No. HY-100523) was obtained from MCE (NJ, USA) and dissolved in dimethyl sulfoxide (DMSO) sourced from Solarbio, Beijing, China. The experimental procedures were conducted in accordance with the manufacturer's protocols.

### R. Statistical analysis

All data are presented as mean ± standard error of the mean (SEM). A two-sided Student's t-test was employed for comparisons between two groups, while one-way analysis of variance (ANOVA) was utilized for comparisons among multiple groups. Pearson correlation coefficients were calculated to evaluate the relationships between data pairs. Statistical analyses were performed using GraphPad Prism 9.0.0, with a significance threshold set at p < 0.05.

## SUPPLEMENTARY MATERIAL

See the supplementary material for the following: GO and KEGG enrichment analysis (Fig. S1); prediction of transcription factors (Fig. S2); quality control and marker selection (Fig. S3); validation of animal model (Fig. S4); immunohistochemistry analysis of REEP1 and ADAD2 expression (Fig. S5); validation of *in vivo* knockdown efficiency of AAV-sh-CYBA and its impact on serum lipid levels (Fig. S6); data collections utilized in this research (Table S1); primer sequences (Table S2); and the details of the five hub genes (Table S3).

## Data Availability

The data that support the findings of this study are openly available in Gene Expression Omnibus at https://www.ncbi.nlm.nih.gov/geo/, Ref. 59.
